# NADPH oxidases in traumatic brain injury – Promising therapeutic targets?

**DOI:** 10.1016/j.redox.2018.03.005

**Published:** 2018-03-15

**Authors:** Merry W. Ma, Jing Wang, Krishnan M. Dhandapani, Ruimin Wang, Darrell W. Brann

**Affiliations:** a Department of Neuroscience and Regenerative Medicine, Medical College of Georgia, Augusta University, Augusta, GA, USA; b Department of Neurosurgery, Medical College of Georgia, Augusta University, Augusta, GA, USA; c Department of Neurobiology, North China University of Science and Technology, Tangshan, Hebei, China

**Keywords:** CCI, Controlled cortical impact, NOX, NADPH oxidase, ROS, reactive oxygen species, TBI, traumatic brain injury, WT, wild-type, NADPH oxidase, NOX, NOX2, NOX4, NOX1, Traumatic brain injury, TBI, FPI, CCI, Controlled cortical impact, CHI, ROS, Oxidative stress, Microglia, Apocynin, gp91ds-tat

## Abstract

Traumatic brain injury (TBI) is a major cause of death and disability worldwide. Despite intense investigation, no neuroprotective agents for TBI have yet translated to the clinic. Recent efforts have focused on identifying potential therapeutic targets that underlie the secondary TBI pathology that evolves minutes to years following the initial injury. Oxidative stress is a key player in this complex cascade of secondary injury mechanisms and prominently contributes to neurodegeneration and neuroinflammation. NADPH oxidase (NOX) is a family of enzymes whose unique function is to produce reactive oxygen species (ROS). Human post-mortem and animal studies have identified elevated NOX2 and NOX4 levels in the injured brain, suggesting that these two NOXs are involved in the pathogenesis of TBI. In support of this, NOX2 and NOX4 deletion studies have collectively revealed that targeting NOX enzymes can reduce oxidative stress, attenuate neuroinflammation, promote neuronal survival, and improve functional outcomes following TBI. In addition, NOX inhibitor studies have confirmed these findings and demonstrated an extended critical window of efficacious TBI treatment. Finally, the translational potential, caveats, and future directions of the field are highlighted and discussed throughout the review.

## 1 Introduction

Traumatic brain injury (TBI) is the leading cause of death and disability in young adults in the United States [1]. It accounts for approximately 2.8 million annual visits to the emergency department, hospitalizations, and deaths [2], [3], [4]. In recent years, TBI has gained recognition as an acute trauma that can progress into a chronic disorder [5], [6]. For instance, TBI in military veterans has been associated with a 60% increase in the risk of dementia [7]. Furthermore, a growing body of evidence suggests that even mild TBI can have detrimental cognitive consequences in the long-term [3]. Unfortunately, the heterogeneity of trauma based on injury location and severity, as well as patient age and associated comorbidities, has posed a significant challenge in the development of effective therapies for TBI [8]. As a consequence, current neurocritical care still focuses primarily on prevention of TBI and stabilizing TBI patients [9].

Further complicating the search for appropriate TBI treatments is the fact that a complex cascade of secondary injury mechanisms develops following the primary mechanical injury [10], [11], [12], [13]. These secondary pathological mechanisms include edema, ischemia, neuroinflammation, and hypoxia. They evolve over minutes to years after the injury, and can ultimately lead to neuronal cell death (even extending into the initially healthy surrounding tissue) [14], [15], [16], [17], [18], [19], [20], [21], [22], [23], [24], [25]. A number of clinical trials have attempted to target some of these pathological mechanisms identified in preclinical animal studies. However, these trials have collectively failed, possibly due to a lack of variety in animal models utilized, rigor and reproducibility across species, or suboptimal drug administration in the trials [26], [27], [28]. Hence, there remains a strong need for rigorous animal studies to understand other injury mechanisms following TBI, which hopefully can lead to effective therapeutic targets for brain injury.

### 1.1 Oxidative stress in TBI

In recent years, there has been growing evidence that oxidative stress contributes significantly to secondary injury in TBI pathology. For instance, many studies have shown that oxidative stress plays a key role in the development of cerebral edema, inflammation, and the secondary neuronal damage found post-TBI [5], [11], [29], [30], [31], [32], [33]. Although reactive oxygen species (ROS) have vital physiological functions [34], [35], [36], pathological conditions, such as brain injury, can quickly shift the ROS/antioxidant balance in favor of ROS and significantly impact the severity and progression of TBI [5], [8], [37]. Since previous animal studies of promising ROS scavengers and ROS degrading agents in other neurodegenerative disorders have failed to translate to the clinic [38], [39], it has been suggested that targeting the *generation* of ROS may be a more successful avenue of therapy for brain injury [5]. Of the many enzymes that produce ROS in the cell, nicotinamide adenine dinucleotide phosphate oxidase (NADPH oxidase; NOX) is the only family of enzymes with the sole purpose of producing ROS, whereas others generate ROS as a byproduct [40], [41]. While NOX enzymes undoubtedly contribute to physiological functions in the brain [42], [43], many laboratories have focused on enhancing our understanding of their pathological role in brain injury [5], [44], [45]. With evidence suggesting that chronic activation of NOX is detrimental and can even exacerbate the primary injury [6], NOX enzymes have emerged as a potential therapeutic target for TBI.

### 1.2 The NADPH oxidase enzymes

**Fig. 1:**
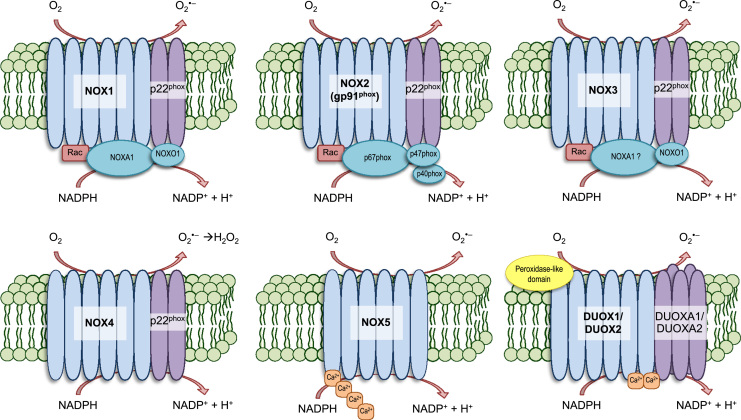
**Structure of active NOX and DUOX enzymes.** NOX and DUOX enzymes have a primary function to generate ROS. Several components make up the active transmembrane enzymes of each NOX/DUOX isoform. NOX1-5 and DUOX1-2 are shown here. NOX 1-3 are the most structurally similar, each requiring cytosolic subunits for activation. It is believed that the NOX4 isoform is constitutively active, yet inducible, and its generated superoxide is rapidly converted into hydrogen peroxide. NOX5 and the DUOX enzymes are reportedly sensitive to cellular Ca^2+^ concentrations. Though not pictured, activation of NOX isoforms may require phosphorylation of different sites within each subunit.

Initially discovered and characterized as the ROS-generating enzyme in phagocytes responsible for the “respiratory burst,” NOX enzymes consume oxygen to generate superoxide and hydrogen peroxide that can go on to produce other forms of ROS, such as hydroxyl and peroxynitrite [43], [44], [46], [47]. To date, seven transmembrane isoforms of the NOX enzyme (Fig. 1) have been identified in non-phagocytic cells, each having binding sites for heme, FAD, and NADPH [48], [49], [50], [51], [52], [53]. NOX1–5 and dual oxidase (DUOX) 1–2 are distributed broadly throughout various tissues and cells, but often times a single isoform is heavily concentrated in specific tissues [44]: NOX1 in the colon [54], [55], NOX2 in phagocytes [56], NOX3 in the inner ear [57], [58], NOX4 in the kidneys [59], [60], NOX5 in the spleen and testis [61], [62], and DUOX1/2 in the thyroid [49], [63]. NOX isoforms are expressed in various brain regions (forebrain, midbrain and hindbrain) and cell types (neurons, astrocytes, and microglia) [44], [64]. Activation of NOX/DUOX enzymes may include several steps involving phosphorylation and translocation of cytosolic subunits, if required, to the membrane where they join transmembrane subunits to form the active complex that transfers an electron from NADPH to O_2_, producing superoxide [44], [48], [49], [50], [51], [52], [53], [65], [66], [67]. Ma et al. summarizes the expression of NOX isoforms in different brain regions and their involvement in brain injury and neurodegenerative diseases [5]. The most studied and heavily implicated isoform in the context of TBI is NOX2. In addition, recent studies also support an emerging role for NOX4 [5], [31]. Unfortunately, not every isoform has been extensively characterized and studied in the pathogenesis of TBI, but the current existing literature supports the potential translation of NOX targeting therapies for treatment of TBI, as will be discussed in the subsequent sections below.

## 2 Elevated expression of NOX enzymes in TBI

### 2.1 Clinical correlations

Several groups have examined the role of NOX isoforms in human TBI pathology, and the clinical and post-mortem human data support NOX involvement in TBI. In humans, TBI increases the expression of NOX2 in circulating monocytes 1 day post-injury (dpi), suggesting that TBI can induce systemic inflammatory responses [68]. Sampling the cerebral cortex from post-mortem human brains revealed peak NOX2 expression in neurons and astrocytes between 6 and 24 h post-injury and peak NOX4 expression in neurons between 1 and 2 dpi [69], [70]. This increased level of NOX2 was associated with increased DNA oxidation [70]. Furthermore, higher expression of NOX2 and NOX4 in the brain correlated with increased severity of TBI as measured by the Glasgow coma scale [69]. Another study using TBI post-mortem human brain samples reported that NOX2 expression was also detected in microglia [70]. Finally, cortices from athletes diagnosed with chronic traumatic encephalopathy also showed higher expression of NOX4 and p22^phox^ (a subunit of NOX 1–4 isoforms) [71].

### 2.2 Animal studies – NOX2

**Fig. 2:**
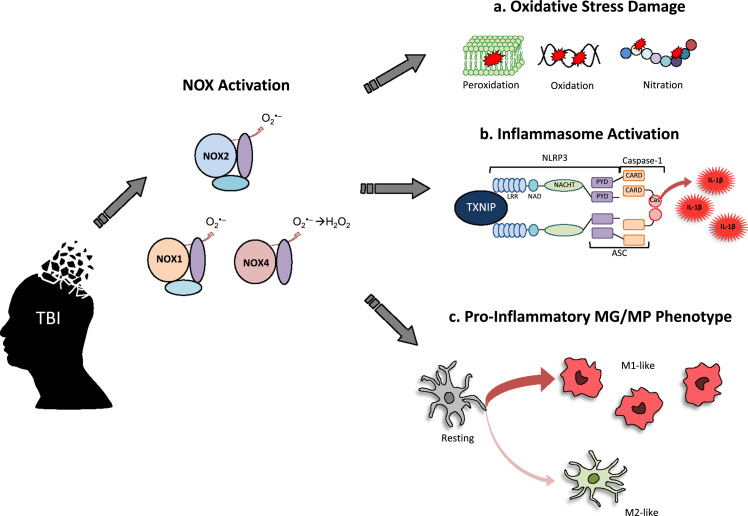
**NOX involvement in secondary TBI pathology**. TBI induces activation of NOX enzymes (in particular, NOX1, NOX2, and NOX4) to produce ROS. The activated NOX induces secondary TBI pathology that can exacerbate the primary injury. A few prominent examples are depicted here: **a.** production of oxidative stress damage (peroxidation of lipids, oxidation of DNA, nitration of amino acids), **b.** generation of pro-inflammatory cytokines (IL-1β) by a TXNIP-mediated activation of NLRP3 inflammasome, and **c.** regulation of MG/MP phenotype towards the pro-inflammatory M1-like phenotype. *Abbreviations: NLRP3 – nucleotide oligomerization domain (NOD)-like receptors containing Pyrin domain-3; TXNIP – thioredoxin interacting protein; LRR – leucine rich repeats; NAD – NACHT-associated domain; NACHT - domain present in NAIP, CIITA, HET-E and telomerase associated protein; PYD – pyrin domain; CARD – caspase recruitment domain; Cas – caspase-1; ASC - apoptosis associated speck-like protein containing a CARD; IL-1β – interleukin-1 beta; MG/MP – microglia/macrophage.*

Animal studies have extensively evaluated NOX elevation and associated pathology following TBI across rodent species and various models of focal and diffuse brain injury. Increases in NOX expression post-injury, which is exacerbated in aged mice [72], are frequently accompanied by evidence of increased ROS production, oxidative stress damage, lesion volume, and cell death that persist throughout the first year following TBI [6], [73], [74], [75], [76], [77]. Of all the NOX isoforms, NOX2 is the most studied in TBI and is found mostly in neurons and microglia following injury [78]. Several groups have shown that TBI-induced NOX2 is associated with increased oxidative damage [77], neuroinflammation [79], and microglial activation (Fig. 2) [77], [80], [81], [82].

Our laboratory and others demonstrated that NOX activity and NOX2 expression in the cortex and hippocampal CA1 region increases rapidly following controlled cortical impact (CCI) with an early peak at 1 h post-injury and a secondary peak from 1 to 4 dpi [77], [83]. Others have characterized the mRNA expression, protein expression, and activity of NOX2 post-TBI in mice and rats and found NOX2 expression and NOX activity to be increased at 1 dpi [72], [73], [74], [76], [78], [84], [85]. Furthermore, translocation of other NOX isoform subunits to the plasma membrane has also been demonstrated for p47^phox^, p67^phox^, and p40^phox^ in the injured brain as early as 6 h post-injury [76], [86], which further supports NOX activation early after TBI. NOX2 activation persists at 7 dpi, when the majority of NOX2 and p22^phox^ expression is found in Iba1^+^ microglia near the lesion [72], [78], [87]. It has even been suggested that NOX activity increases in the contralateral cortex at 7 dpi [73], indicating that global brain inflammation can occur at this time-point. Microglia that persist at 1 month after TBI in the injured hemisphere highly express NOX2 [78], [81]. Furthermore, an acute injury such as TBI can lead to chronic activation of microglia, and their elevated expression of NOX2 at 3–4 months [72], [88] and even 1 year after the initial injury [6]. This finding suggests that NOX2 may play a role in the chronic inflammation that occurs following TBI.

Interestingly, a series of recent studies demonstrated that microglia shift toward a pro-inflammatory state (M1-like) that express high levels of NOX2 at 7 dpi, which is associated with increased cortical and hippocampal neurodegeneration [82], [89]. It's worthwhile to note that NOX2 is also highly activated in infiltrating macrophages following TBI, suggesting the possibility of peripheral involvement in TBI pathology [90]. Though some anti-inflammatory (M2-like) microglia are present at 7 dpi, they are reduced by 5 weeks and reportedly absent at 12 weeks and 1 year post-TBI [6]. Since the generally M1-like microglia isolated from the injured mouse cortex at 7 dpi are neurotoxic [82], it's strongly possible that these NOX2-expressing microglia contribute to the progressive lesion expansion seen in TBI at chronic time-points. In support of this possibility, microglia isolated from NOX2^-/-^ mice after TBI had a significantly attenuated neurotoxicity on surrounding neurons [82]. This reduced neurotoxicity could be due to reduced generation of damaging cytokines, as NOX2^-/-^ mice had a profoundly attenuated expression of TNF-α and IL-1β [82]. Taken as a whole, these animal studies demonstrate that NOX2 is upregulated in the injured cortex after TBI, and thus could play an important role in TBI pathogenesis and post-injury neuroinflammation.

### 2.3 Animal studies – other NOX isoforms

Although the majority of TBI studies have focused on NOX2, other isoforms have also been examined, and their expression has been characterized in the post-TBI brain [78]. Of the ones studied, NOX3 appears unresponsive to injury, showing little change in its neuronal expression after TBI [78]. Several studies have investigated involvement of NOX1 in TBI, showing that an increased NOX activity at 1 dpi was accompanied by increased NOX1 expression [75], [91], [92]. At this early time-point, the increased NOX1 expression was accompanied by evidence of elevated oxidative damage, such as lipid peroxidation and protein nitration [75], [91], [92]. NOX1 is induced after both single and repeated blast injuries, with NOX1 mRNA upregulated as early as 6 h post-injury, followed by a peak NOX1 protein expression at 2–8 dpi [91]. TBI-induced NOX1 correlates with instability of the blood brain barrier (BBB) in the cerebral microvessels, suggesting that NOX1 is associated with cerebrovascular injury [91].

Reports of NOX4's involvement in TBI have also emerged recently. NOX4 has been characterized in neurons, astrocytes, and microglia following CCI [78]. A recent study from our group found that NOX4 mRNA was significantly upregulated between 1 and 7 dpi, returning to baseline by 14 dpi [93]. Furthermore, Western blot analysis showed elevated NOX4 protein expression at 1, 4, and 7 dpi, and double immunohistochemistry revealed that the elevated NOX4 expression was predominantly located in neurons in the injured cerebral cortex [93]. The elevated NOX4 expression in the cerebral cortex after TBI was correlated with increased oxidative stress damage to DNA, protein and lipids [93]. Likewise, in blast-injured rats, elevated NOX4 and p22^phox^ expression correlated with increased superoxide production [71]. Finally, NOX5 and the DUOXs have not been characterized in TBI, and thus studies to explore their potential role in TBI pathogenesis are needed.

## 3 Evidence from NOX knockout studies

Perhaps the most definitive evidence we have that implicates a causative role of NOX isoforms in TBI pathology comes from studies utilizing NOX knockout mice. The first report utilizing NOX2^-/-^ mice (gp91^-/-^ mice) in brain injury employed a surgically induced brain injury (SBI) model [94]. Although edema was unaltered by the deletion of NOX2, knockout mice had improved neurological scores following injury as compared with wild-type mice in SBI [94]. In classic models of TBI, such as CCI, the first report of NOX2^-/-^ mice showed reduced contusion area and apoptosis following TBI that was coupled with a reduction in superoxide and peroxynitrite metabolites in the injured cortex [80]. In addition to decreasing lesion volume, deletion of NOX2 improved motor coordination at 7 [95], 14, and 21 dpi [90], indicating that targeting NOX2 may be functionally beneficial. Knockout studies have also confirmed an extensive role of NOX2 in TBI-induced neuroinflammation. For instance, NOX2^-/-^ mice have significantly reduced NLRP3 inflammasome expression and activation in the injured cortex following TBI [79]. This effect in NOX2^-/-^ mice may be explained by a correlated decrease of TBI-induced thioredoxin-interacting protein (TXNIP), which has been implicated to directly link oxidative stress to NLRP3 inflammasome activation [79].

Interestingly, deletion of NOX2 also reduced expression of M1-like markers in microglia/macrophages (MG/MP) in the injured cerebral cortex without altering total number of infiltrating macrophages (CD45^hi^ cells) to the injury site [90]. The shift in MG/MP polarization dynamics towards the M2-like phenotype in injured NOX2^-/-^ mice, which may be a result of enhanced IL-10 and STAT3 signaling [96], was also associated with decreased production of pro-inflammatory cytokines via a down-regulation of the classical NF-κB pathway [82]. As mentioned previously, microglia isolated from NOX2^-/-^ injured mice had attenuated neurotoxicity in cultured neurons [82], reinforcing the notion that NOX2 inhibition is neuroprotective following TBI.

In addition, deletion of NOX4 was also found by our group to attenuate the severity of TBI via reduction of lesion size, oxidative damage, neurodegeneration, and apoptosis [93]. This finding indicates that in addition to NOX2, NOX4 also plays a significant role in the pathology and neurodegeneration that occurs in the injured cortex after TBI. These animal studies utilizing NOX knockouts have been summarized in Table 1. To date, no studies utilizing NOX1^-/-^ or NOX3^-/-^ mice have been conducted in TBI. In addition, development of NOX2/4 double knockout models may be useful for evaluating potentially additive effects of these isoforms in TBI. Finally, cautious interpretation of the knockout animal studies discussed above is necessary due to the global knockout approach, which may have life-long developmental and compensatory confounds in addition to a secondary effect.

**Table 1:** **Effect of NOX deletion in models of TBI.** Overview of published studies utilizing genetic NOX knockout mice in models of TBI. The animal TBI model used, the specific region of interest (ROI) evaluated, and the major findings of these studies have been summarized. To date, only NOX2 and NOX4 knockout mice have been studied. *Abbreviations: SBI – surgical brain injury; CCI – controlled cortical impact*.

**NOX modulation**	**Animal model**	**ROI**	**Results**	**Ref.**
**NOX2**^**-/-**^	SBI	Frontal lobe	• ↑ Neurological outcomes • No change in edema	[94]
**NOX2**^**-/-**^	CCI	Cortex	• ↓ Lesion size, apoptosis, oxidative stress	[80]
**NOX2**^**-/-**^	CCI	Cortex	• ↓ Clic1 and CD68+ MG/MP, lesion volume, neurodegeneration • Mitigates M1-like and promotes M2-like response in MG/MP • ↑ Motor coordination • ↑ IL-4Rα in infiltrating MP	[90]
**NOX2**^**-/-**^	CCI	Cortex	• ↓ Lesion size, neuronal damage, NLRP3 inflammasome activation, IL-1β	[79]
**NOX2**^**-/-**^	CCI	Cortex, CA1 hippocampus	• ↓ NFκB activation and in MG/MP, oxidative stress, lesion size, apoptosis, MG/MP neurotoxicity • Shifts MG/MP toward M2-like polarization	[82]
**NOX2**^**-/-**^	CCI	Cortex	• ↑ Motor function • ↓ Lesion volume	[95]
**NOX4**^**-/-**^	CCI	Cortex	• ↓ Neuronal oxidative stress, lesion volume, neurodegeneration, apoptosis	[93]

## 4 Evidence from NOX inhibition studies

To circumvent the limitations of global knockout approaches, the role of NOX enzymes have been further studied through utilizing NOX inhibitors to investigate whether acute inhibition of NOX can offer similar benefits following TBI. One of the most studied NOX inhibitors in TBI research is apocynin, a compound isolated from the medicinal plant *Picrorhiza kurroa* that inhibits NOX2 via preventing the membrane translocation of p47^phox^ and p67^phox^ [97], [98], [99], [100]. Apocynin has also been reported to reduce oxidative damage by scavenging hydrogen peroxide and hypochlorous acid in phagocytic cells [101], [102].

Administration of apocynin, in doses varying from 5 mg/kg to 100 mg/kg, has been documented to decrease NOX2 expression, ROS production, and oxidative damage in animal models of TBI [74], [83], [103], [104]. In animal models of diffuse brain injury via weight drop, pre-treatment with apocynin resulted in neuroprotection of the cortex [77], [83] and hippocampus [77], [103], reduced edema [83], [103], [104], and attenuated neuroinflammation [77], [103] while improving TBI-induced neurological deficits [83], [104]. Further supporting NOX2's prominent role in TBI pathology, gp91ds-tat, a competitive inhibitor specific to NOX2, was also shown to be neuroprotective following TBI [77]. These pre-injury inhibition studies demonstrate that acute targeting of NOX can be beneficial in similar ways to long-term, global knockdown of NOX2. However, the post-injury administration of any potentially therapeutic agent is critical for future clinical application. While investigating a therapeutic role of NOX inhibition in TBI, several groups discovered that the post-TBI administration of apocynin can also decrease NOX2 expression and accompanied oxidative stress in rodents [77], [82], [105]. Administration of apocynin, at a dose as low as 5 mg/kg, within two hours of injury is neuroprotective [77], [106], as shown by attenuated lesion volume [105], reduced neuroinflammation within the first week of injury [77], [106], and decreased expression of Alzheimer's disease (AD)-related proteins [77]. While initial assessment of edema and neuromotor deficits within the first 24 h is conflicted [105], extended assessment found reduced edema within 7 dpi [106], attenuated memory impairment at 7 dpi [105], and improved sensorimotor performance at 7, 14, and 21 dpi [95], [106], [107]. Intriguingly, delayed administration of apocynin at 24 h post-injury also attenuated oxidative damage, reduced NLRP3 inflammasome activation, and promoted microglial polarization towards the M2-like phenotype following TBI in the mouse [79], [82]. A delayed administration of gp91ds-tat at 24 h post-injury was also able to attenuate cognitive deficits [90], further suggesting that the critical window of TBI treatment can be extended to 24 h post-injury. These animal studies utilizing NOX inhibitors and their dosing regimens are summarized in Table 2.

**Table 2:** **Effect of NOX inhibition in models of TBI.** Overview of published studies utilizing pharmacological inhibition of NOX enzymes in models of TBI. The dosing regimen of well-cited NOX inhibitors, the model of TBI used, the specific region of interest (ROI) evaluated, and the major findings of each study have been summarized. *Abbreviations: SBI – surgical brain injury; CHI – closed-head injury; CCI – controlled cortical impact; mLFPI – moderate lateral fluid percussion injury; MG/MP – microglia/macrophage*.

**NOX modulation**	**Animal model**	**ROI**	**Results**	**Ref.**
**Apocynin 5** **mg/kg ip; 30** **min pre-SBI**	SBI	Frontal lobe	• No change in neurological outcome, edema • ↓ Oxidative stress at 3 h; not at 24 h	[94]
**Apocynin 50** **mg/kg ip; 30** **min pre-TBI**	Weight drop (CHI)	Injured hemisphere; cortex	• ↓ Edema, neurological deficits, oxidative stress, apoptosis	[83]
**Apocynin 4** **mg/kg ip; 20** **min pre-TBI**	CCI	Cortex; CA1 /CA3 hippocampus	• ↓ Microglial activation, β-amyloid, neuronal death	[77]
**Apocynin 100** **mg/kg ip; 15** **min pre-TBI**	Weight drop (open skull)	CA3 hippocampus	• ↓ Neurodegeneration, oxidative stress, BBB disruption, microglial activation	[103]
**Apocynin 10** **mg/kg ip; 5** **min or 2** **h + 2nd dose at 24** **h post-TBI**	CCI	Cortex	• ↑ Motor function • No change in lesion volume	[95]
**Apocynin 5** **mg/kg sc; 30** **min and 24** **h post-TBI**	mLFPI	Cortex	• ↓ Memory impairment, IL-1β, oxidative stress, lesion volume • No change in neuromotor deficits, edema	[105]
**Apocynin 4** **mg/kg ip; 2** **h post-TBI**	CCI	Cortex; CA1 /CA3 hippocampus	• ↓ Microglial activation, β-amyloid, neuronal death	[77]
**Apocynin 5** **mg/kg ip; 23** **h post-TBI, qd**	CCI	Cortex, CA1 hippocampus	• ↓ NFκB activation in MG/MP, oxidative stress, lesion size, apoptosis, MG/MP neurotoxicity • Shifts MG/MP toward M2-like polarization	[82]
**Apocynin 5** **mg/kg ip; 23** **h post-TBI, qd**	CCI	Cortex	• ↓ NLRP3 inflammasome activation, IL-1β	[79]
**Gp91ds-tat; 250 μg/mouse ip; 20** **min pre-TBI**	CCI	Cortex	• ↓ Edema • ↑ Neuronal density	[77]
**Gp91ds-tat; 5** **mg/kg ip; 24** **h, 48** **h, and 72** **h post-TBI**	CCI	Cortex	• ↓ CD16/32 and ↑ TGFβ expression in cortex • ↓ Oxidative stress • Shifts MG/MP toward M2-like polarization	[89]
**Gp91ds-tat; 5** **mg/kg ip; 1d, 2d, and 3d post-TBI**	CCI	Cortex	• Promoted M2-like activation of MG/MP • ↑ Spatial working memory • No change in motor coordination	[90]

Mechanistically, current evidence strongly suggests that the beneficial effects of targeting NOX may be mediated in the injured brain through attenuated neuroinflammation and a shift in cellular dynamics towards an anti-inflammatory state [79], [82], [89], [90], [96], [106]. Inhibition of NOX decreased TLR4 and NF-κB signaling and reduced expression of pro-inflammatory cytokines [82], [106]. Similar to NOX2^-/-^ studies [82], [90], [96], NOX inhibition also enhances the M2-like phenotype of MG/MP in the injured cortex after TBI [82], [89], [90]. The benefit of NOX targeting appears to reach beyond reduction of oxidative damage and may be key in limiting chronic neuroinflammation following TBI by reducing microglia activation in the injury site and altering MG/MP inflammatory phenotypes.

## 5 Therapeutic considerations

### 5.1 Targeting NOX2

As described above, NOX2 targeting via selective inhibitors or via apocynin has been reported to improve cognitive outcomes following TBI in animal models [83], [90], [95], [105]. In particular, apocynin is attractive for use in TBI treatment due to its ability to reach the brain parenchyma with oral administration, high stability, and low general toxicity even at much higher doses than that used in TBI studies [108]. Apocynin has also been used in clinical trials of inflammatory respiratory conditions and showed efficacy with no adverse effects [109], [110], [111]. Apocynin's broad range of potential therapeutic application across different tissues, such as arthritis [112], renal ischemia [113], vascular disease [114], and chronic obstructive pulmonary disease [115], may cause concern for potential off-target effects of systemically administering apocynin. Although systemic administration of apocynin can limit the extent of TBI, a targeted approach to deliver apocynin to a limited tissue area or organ may be optimal to limit suppression of physiological NOX signaling. Cell-specific targeting of NOX inhibitors, towards activated pro-inflammatory microglia for example, may also provide yet another approach that would limit off-target effects.

Along these lines, the most isoform-selective NOX inhibitor to date, gp91ds-tat (also known as NOX2ds-tat) is a synthetic, small peptide inhibitor that was designed to penetrate cells and prevent the assembly of the active NOX2 complex by inhibiting the association of p47^phox^ with gp91^phox^ [116]. As previously mentioned, several groups have treated injured animals with gp91ds-tat and reported beneficial effects. However, as a peptide, gp91ds-tat has low oral bioavailability and low stability [117], which may hinder its translation to clinical applications. It is also uncertain whether this small peptide can pass through the BBB in mild injuries that may leave the BBB intact. Nonetheless, its high selectivity for NOX2 [118] is attractive for studying NOX2-specific mechanisms underlying TBI pathology.

### 5.2 Targeting NOX1 and NOX4

The majority of studies presented in this review focus on the role of NOX2 in mediating neuroinflammation and oxidative stress after TBI, with few recent studies implicating a role for NOX1 and NOX4. Although NOX2 seems the obvious choice for translational pursuit, targeting NOX4 may be advantageous if specific inhibitors are developed. Hospitalized TBI patients have increased susceptibility to potentially fatal nosocomial infections [119], [120], [121], [122]. NOX2^-/-^ mice are frequently used to model chronic granulomatous disease, an inherited disorder characterized by recurrent infections due to defects in innate immunity [123], [124], [125]. Considering the elevated risk of infection in TBI patients, NOX2 targeting could potentially exacerbate susceptibility to infections post-hospitalization, though further studies are necessary to evaluate this potential disadvantage. In contrast, NOX4^-/-^ mice are generally healthy and lack gross phenotypes [126], [127], and NOX4 is primarily upregulated in neurons following TBI [93]. These characteristics position NOX4 to be a promising novel target in the treatment of TBI that may avoid potential immune suppression of NOX2-expressing microglia. Furthermore, whether apocynin exerts some effect via inhibition of NOX4 remains unclear. Although apocynin-mediated neuroprotection was lost in NOX2^-/-^ mice [128], similar studies have not been conducted with NOX4^-/-^ mice.

While there is no NOX4-specific inhibitor, a new NOX1/NOX4 dual inhibitor GKT137831 has been developed, which may be worth investigating in TBI. GKT137831 has already been shown to reduce neuronal death and degeneration in a rat model of subarachnoid hemorrhage [129]. Furthermore, orally bioavailable GKT137831 has already been employed in a phase II clinical trial for diabetic nephropathy and primary biliary cholangitis, and appears to be well tolerated with no adverse events (Clinical Trial ID: NCT02010242, NCT03226067). Based on these observations, preclinical studies to evaluate the efficacy of GKT137831 in treatment of TBI appear to be needed, as they would help determine the therapeutic potential of targeting the NOX1 and NOX4 isoforms in TBI pathology.

### 5.3 Additional considerations and future directions

An important caveat in experimental animal studies on TBI is that the vast majority of animal TBI studies have been conducted in adult male mice of approximately 3 months of age. The lack of age range in the studies is a concern and potentially limiting in translating these basic findings therapeutically to humans. For instance, the highest rates of TBIs are amongst the oldest or youngest age groups who are particularly prone to falling [4]. Thus, NOX involvement should continue to be evaluated to include juvenile and aged mice, especially since one report suggests that aged mice appear to have prolonged NOX expression and worse outcomes after TBI [72]. In addition, as NOX activity and function may vary with gender [130], [131], [132], [133], further pre-clinical studies should evaluate whether NOX inhibition is similarly efficacious in female mice. As TBI is an epidemic that impacts males and females throughout all life stages, perhaps further studies of NOX involvement, and its potential therapeutic targeting should be explored across all ages and genders.

In addition, most animal studies have focused on acute time frames after TBI (hours to days), and there is a paucity of chronic long-term studies after TBI (e.g. months to years after TBI). This is important, because activated MG/MP are present up to 18 years following a single TBI in the human brain [134], and sustained inflammation over the initial year post-TBI is predictive of global outcome at 6 and 12 months post-TBI [135]. Animal studies have suggested that chronically upregulated neuroinflammation may contribute to the progressive nature of severe TBI [6]. In the long-term, acute inflammatory processes can become self-perpetuating, leading to sustained neuroinflammation that is detrimental [136], [137]. Both NOX2 knockout and inhibition studies described in the above sections have demonstrated strong anti-inflammatory results, presumably via regulation of MG/MP phenotypes. Whether the suppressed neuroinflammation obtained from acutely targeting NOX in animal models can be sustained past the initial weeks following TBI remains unknown, as studies with knockout mice or inhibitors have not extended to these far time-points. Including long-term chronic endpoints in future inhibitor and NOX deletion studies will be critical to resolving this question.

## 6 Concluding remarks

Despite an abundance of successful pre-clinical studies, the search for an acute neuroprotective drug has yet to deliver promising treatments for TBI. These failures stress the need for innovative therapeutic targets. Though there is significant evidence that NOX enzymes are involved in TBI pathology, many challenges still exist in this pre-clinical phase. Growing the supporting evidence for NOX inhibition across different species, genders, ages, assessment time-points, drug doses, injury sites, and injury models while remaining cognizant of negative data will aid the evaluation of NOX as a target for TBI.
